# Aging with Purpose: The Impact of Subjective Aging

**DOI:** 10.1093/geroni/igaf122.3700

**Published:** 2025-12-31

**Authors:** Yiqing Yang, Kelin Li

**Affiliations:** Western Carolina University, Cullowhee, North Carolina, United States; California State University Dominguez Hills, California State University Dominguez Hills, California, United States

## Abstract

Subjective aging is a well-established predictor of health and well-being in older adults, while a strong sense of purpose in life (PIL) has consistently been linked to positive health outcomes. Despite PIL’s role as a core dimension of eudaimonic well-being, its relationship with subjective aging remains underexplored. This study examines the correlates of PIL, measured using Ryff ‘s 7-item Purpose in Life Subscale, with a focus on two widely used subjective aging measures among 150 community-dwelling older Americans. Spearman correlation indicates that age, gender, race, and partner status are not significantly correlated with PIL. Ordinary least squares regression analyses reveal that felt age and self-perceptions of aging, assessed via an 8-item Attitudes towards Own Aging subscale, are significant predictors of PIL. In the overall model (R² = 0.50, F (12, 137) = 11.62, p < 0.0001), both measures of subjective aging remain significant after adjusting for education, financial strain, religiosity, self-rated health, and depressive symptoms. Additionally, higher educational attainment, greater religiosity, and fewer depressive symptoms are significantly associated with higher PIL, while financial strain and self-rated health are not. These findings suggest that fostering positive subjective aging perceptions may be a promising target for interventions aimed at enhancing PIL, ultimately promoting healthy and successful aging.

